# Mandibular Reconstruction With the Contralateral Vascularized Iliac Flap Using Individual Design: Iliac Crest Used to Reconstruct the Ramus and the Anterior Border of the Iliac Wing Used to Reconstruct the Inferior Border: A Case Report

**DOI:** 10.3389/fsurg.2022.924241

**Published:** 2022-07-11

**Authors:** Guangcai Xu, Ju Jia, Xuepeng Xiong, Liwei Peng, Lin-lin Bu, Xiqian Wang

**Affiliations:** ^1^ School of Stomatology, Henan University, Kaifeng, China; ^2^ Department of Oral and Maxillofacial Surgery, Henan Provincial People's Hospital; Zhengzhou University People's Hospital; Henan University People's Hospital, Zhengzhou, China; ^3^ Department of Oral Maxillofacial Head Neck Oncology, School and Hospital of Stomatology, Wuhan University, Wuhan, China

**Keywords:** mandibular, vascularized iliac flap, reconstruction, 3D printing, individual

## Abstract

Mandible defects resulting from resection of benign or malignant lesions, trauma, or radionecrosis are commonly encountered in the oral and maxillofacial department. Vascularized bone flaps, in general, provide the best functional and aesthetic outcome. The iliac crest provides a large piece of curved cortico-cancellous bone, measuring 6–16 cm in length. It has a natural curvature that complements the curve of the lateral and sometimes anterior mandible and can be placed accordingly to fill defects. In the paper, we report a mandibular reconstruction with a vascularized iliac flap using individual virtual preoperative planning and 3D printing technology. We want to offer a new design idea for mandibular defect reconstruction.

## Patient

**Figure 1:**
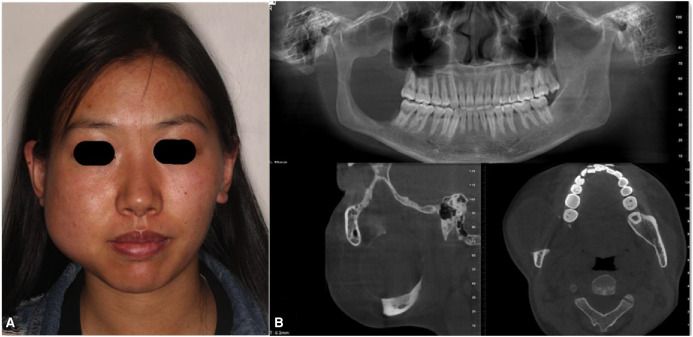
Clinical examination revealing a significant bulge of the right mandibular: cone beam computerized tomography (CBCT) demonstrating that (**A**) there was a bone mineral density reducing area of nabout 4.8 cm × 4.6 cm × 4.9 cm in the right mandible from 47 to the mandibular ramus region and (**B**) Buccolingual bulge was obvious.

A 24-year-old female was admitted to the Department of Oral & Maxillofacial-Head & Neck Oncology, Stomatology Hospital of Wuhan University. The patient found a bulge in the right mandible 10 months ago, and the bulge had been increasing gradually. After admission, clinical examination revealed a significant bulge of the right mandible (Figure 1A). Cone beam computerized tomography (CBCT) demonstrated that there was a bone mineral density reducing area of about 4.8 cm × 4.6 cm ×4.9 cm in the right mandible from 47 to the mandibular ramus region, and a buccolingual bulge was obvious (Figure 1B). The primary diagnosis was a right mandibular ameloblastoma, and the postoperative pathology also showed ameloblastoma.

## Preoperative Planning

**Figure 2:**
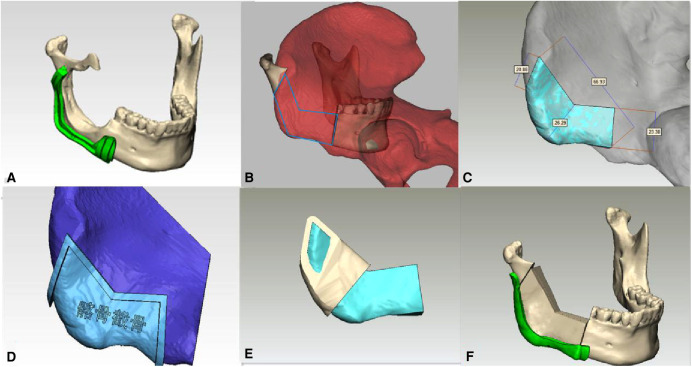
Virtual model created by computer-aided design (CAD); the osteotomy line and surgical cutting guides for the mandible were designed first (**A**); the model of the defect region was used to look for the most matching region in the Iliac bone model, and we found that the most matching region was on the left iliac bone. The anterior superior iliac spine would be used to reconstruct the angle of the mandible, the iliac crest would be used to reconstruct the posterior border of the mandible ramus, and the anterior border of the iliac wing would be used to reconstruct the inferior border of the mandible; the natural curvature of left Iliac bone could complement the curve of the mandible perfectly and can be placed accordingly to fill the defect (**B**); then, the virtual iliac bone graft model was obtained (**C**), which was used to design the bone graft cutting guide (**D**), shaping guide (**E**), and fixation guide (**F**).

**Figure 3:**
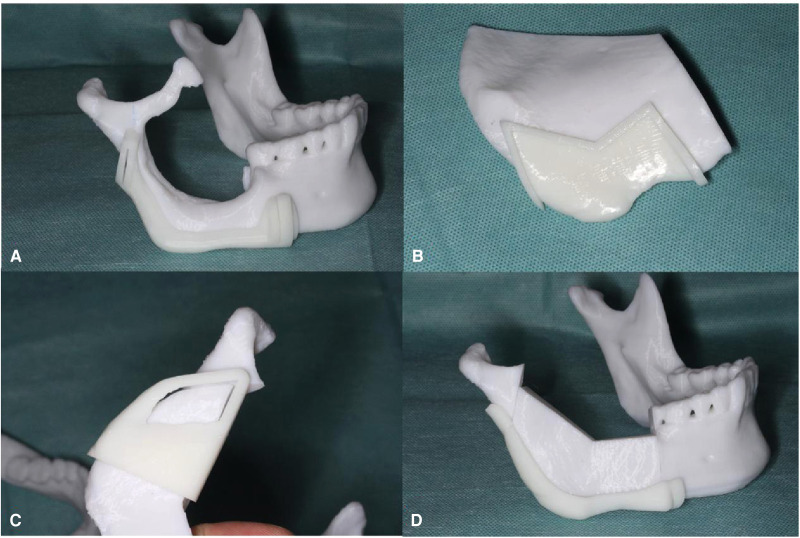
All models and guides were printed on a three-dimensional (3D) printer: original mandible model and cutting guide (**A**); iliac model and cutting guide (**B**); shaping guide (**C**); and reconstructed mandible model and fixation guide (**D**).

The treatment aim was to resect the tumor radically and reconstruct the mandible immediately. Because the defect was less than 9 cm, we decided to reconstruct the defect with a vascularized iliac flap. The CBCT and the iliac bone CT data were obtained, and then, a virtual model by computer-aided design (CAD) was created. The osteotomy line and surgical cutting guide for the mandible were designed first (Figure 2A). The model of the defect region was used to look for the most matching region in the iliac bone model; we found that the most matching region was on the left iliac bone. The anterior superior iliac spine could be used to reconstruct the angle of the mandible, the iliac crest would be used to reconstruct the posterior border of the mandible ramus, and the anterior border of the iliac wing would be used to reconstruct the inferior border of mandible; the natural curvature of left iliac bone could complement the curve of the mandible perfectly and can be placed accordingly to fill the defect (Figure 2B). Then, we obtained the virtual iliac bone graft model (Figure 2C), which was used to design the bone graft cutting guide (Figure 2D), shaping guide (Figure 2E), and fixation guide (Figure 2F). All the models and guides were printed by a three-dimensional (3D) printer (Figures 3A–D).

## Operation

**Figure 4:**
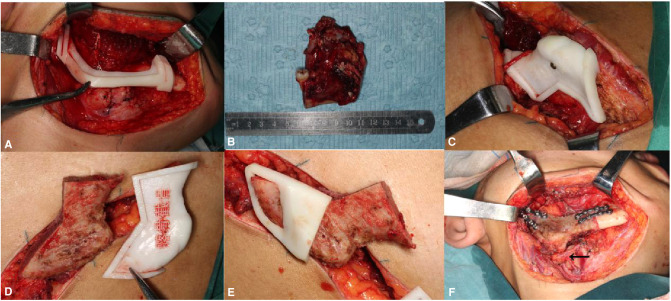
According to the surgical cutting guide, the right mandible from 47 to the condylar neck was resected (**A**,**B**); and the vascularized iliac flap was harvested and prepared (**C–E**). The prepared vascularized iliac flap was fixed to the mandible to reconstruct the mandible defect by a titanium plate according to the fixation guide, and the deep circumflex iliac artery (DICA) and the deep circumflex iliac vein (DICV) were anastomosed with facial artery and external jugular vein; the arrow shows that the vessel pedicle was in a line when the iliac bone flap was transferred to the mandible defect area (**F**).

**Figure 5:**
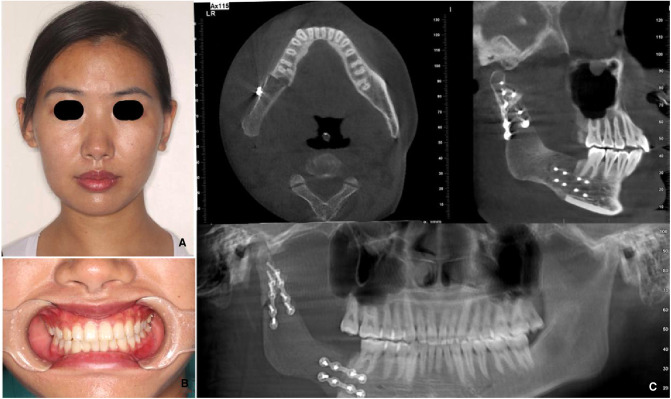
Postoperative anterior view showing that the mandible is symmetrical for 3 months after operation (**A**); postoperative CBCT demonstrating that both the shape of the mandible and the height and width of the alveolar crest are satisfactory (**B**). L stands for the left side, R stands for the right side, both sides are ruler.

**Figure 6:**
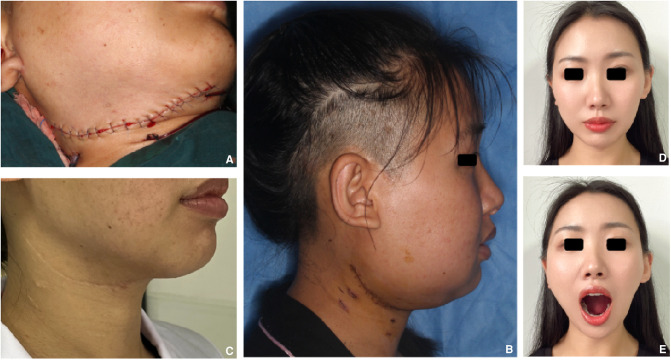
Lateral views showing that the facial incision healed well: (**A–C**) postoperative, 1 week, and 7 years after operation; postoperative function and facial appearance recovered well (**D,E**).

According to the preoperative planning and the surgical cutting guide, the right mandible from 47 to the condylar neck was resected (Figures 4A,B), and the vascularized iliac flap was harvested and prepared (Figures 4C–E). The prepared vascularized iliac flap was fixed to the mandible to reconstruct the mandible defect by a miniplate (Stryker Leibinger Gmbh&CO.CK. Botzinger StraBe 41,7911 Freiburg, Germany) according to the fixation guide. Additionally, the deep circumflex iliac artery (DICA) and the deep circumflex iliac vein (DICV) were anastomosed with the facial artery and external jugular vein, respectively (Figure 4F). The postoperative anterior view showed that the mandible is symmetrical (Figure 5A). Postoperative CBCT demonstrated that the grafted iliac bone matched the shape of the mandible satisfactorily (Figure 5B). The patient was satisfied with the function, facial appearance, and local scar recovery after the operation (Figure 6).

## Discussion

With the development of society, patients’ desire for the result of reconstruction is stronger and higher, both aesthetically and functionality. A vascularized iliac flap is one of the best choices in mandibular reconstruction. The vascularized iliac flap is based on the deep circumflex iliac artery (DCIA), which arises from the lateral aspect of the external iliac artery. The diameter of the DCIA is in the range of 2–3 mm, and the length of the pedicle from the anterosuperior iliac spine to its junction at the external iliac artery is around 5–7 cm (1). The iliac crest provides a large piece of curved cortico-cancellous bone, measuring 6–16 cm in length. The iliac bone flap can provide enough bone height and thickness in mandible reconstruction and osseointegration dental implantation. The natural curvature of the iliac crest makes shaping the bone in anterior defects difficult (2). However, now, with the help of CAD virtual design, surgical cutting and fixation guides, and 3D printing technology, the shaping of bone grafts becomes much easier (3–5).

**Figure 7:**
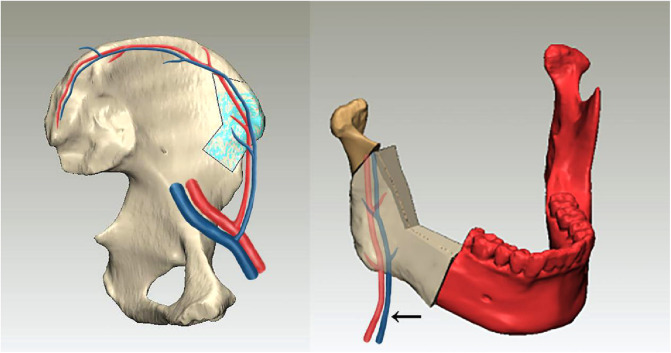
Sketch map showing the relationship between the vessel pedicle and the iliac bone graft; the arrow shows that this design could avoid the twist of the vessel pedicle and keep it in a line when the iliac bone flap was transferred to the mandible defect area.

Generally, the iliac bone flap has a natural curvature that complements the curve of the lateral and sometimes anterior mandible and can be placed accordingly to fill defects. The homolateral iliac is always chosen as the donor site to reconstruct the mandibular defect, with the iliac crest being used to reconstruct the inferior border of the mandible, the anterior superior iliac spine being used to reconstruct the angle of the mandible, and the anterior border of the iliac wing being used to reconstruct the posterior border of the mandible ramus. In this case, however, we found that the most matching region was on the contralateral (left) iliac bone. The natural curvature of the left iliac bone could complement the curve of the mandible perfectly. In addition, this design could avoid the twist of the vessel pedicle and keep it in a line when the iliac bone flap was transferred to the mandible defect area (Figure 7).

## Data Availability

The raw data supporting the conclusions of this article will be made available by the authors, without undue reservation.
